# Appropriateness of Upper Gastrointestinal Endoscopy: Will the Diagnostic Yield Improve by the use of American Society of Gastroenterology Guidelines?

**DOI:** 10.5005/jp-journals-10018-1187

**Published:** 2016-12-01

**Authors:** Muazzam Tahir

**Affiliations:** Department of Surgery and Gastroenterology, Taranaki Base Hospital, Wellington, New Zealand

**Keywords:** American Society of Gastroenterology, Open access, Upper gastrointestinal endoscopy.

## Abstract

**Aim:**

Open access endoscopy allows physicians and general practitioners (GIs) to refer patients for endoscopy without prior outpatient consultation. This system was introduced to reduce waiting time to the procedure and subsequent diagnosis. Concerns have been raised regarding misuse of this system with increasing number of inappropriate referrals and hence more normal examinations, which has implications on a public-funded health system. The aim of this study was to assess the appropriate use of the open access system at a rural New Zealand hospital and to see if the diagnostic yield improves by following the American Society of Gastroenterology (ASGE) guidelines for upper gastrointestinal endoscopy [esophagogastroduodenoscopy (OGD)].

**Materials and methods:**

This was a prospective study including all the patients who had OGD at Taranaki Base Hospital between December 2013 and 2014. A total of 1,019 patients had OGD during this time period. The ASGE guidelines were used to see the relationship between appropriateness of OGD and finding of a relevant endoscopic diagnosis.

**Results:**

Fifty-eight percent of the OGDs were judged to be appropriate and 42% inappropriate by the explicit criteria. No cancer was found in OGDs judged to be inappropriate. Upper gastrointestinal (GI) endoscopies judged appropriate yielded significantly more relevant lesions than those judged to be inappropriate [65% *vs* 32%; odds ratio 3.94, 99% confidence interval (CI) 2.78, 5.57; p < 0.01].

**Conclusion:**

The use of ASGE guidelines increases diagnostic yield of OGDs done, which is crucial to cost-effectiveness of an open access system and makes the system more efficient in selecting and treating patients who need it the most, in an acceptable time span.

**How to cite this article:**

Tahir M. Appropriateness of Upper Gastrointestinal Endoscopy: Will the Diagnostic Yield Improve by the use of American Society of Gastroenterology Guidelines? Euroasian J Hepato-Gastroenterol 2016;6(2):143-148.

## INTRODUCTION

Open access endoscopy system allows physicians to directly schedule common outpatient endoscopic procedures without prior consultation. The increasing reliance by doctors on upper gastrointestinal (GI) endoscopy [esophagogastroduodenoscopy (OGD)] for diagnosis, surveillance, treatment, or exclusion of upper GI diseases has led to an increased demand for OGD.^1^ Due to this increased demand, open access endoscopy system was introduced to expedite examination without the need for prior outpatient appointment. Concerns have been raised repeatedly that this system has increased the overall cost associated with this procedure with an increase in the waiting time.^2,3^ This is believed to be due to an increase in the number of inappropriate referrals with low positive yield.^2^ Hence, adherence to appropriate indications for endoscopic procedures is essential to the rational use of finite resources in an open access system. The guidelines published by the American Society of Gastroenterology (ASGE) has been used to define the appropriateness of numerous indications for OGD (Table 1).^4-8^ The objective of this study was to assess the appropriateness of OGD referrals to a rural New Zealand hospital and to see if the diagnostic yield increases according to the appropriateness of indication, according to the ASGE guidelines. As far as we know, this study is the first of its type in Australasia, where health system is publicly funded and does not have infinite resources.

**Table 1:** **Table 1:** American society of gastroenterology classification of appropriate and not appropriate indications for OGD

A. Upper abdominal symptoms that persist despite an appropriate trial of therapy.	
B. Upper abdominal symptoms associated with other symptoms or signs suggesting structural disease (e.g., anorexia and weight loss) or new-onset symptoms in patients older than 50 years of age.	
C. Dysphagia or odynophagia.	
D. Esophageal reflux symptoms that persist or recur despite appropriate therapy.	
E. Persistent vomiting of unknown cause.	
F. Other diseases in which the presence of upper GI pathology might modify other planned management. Examples include patients who have a history of ulcer or GI bleeding who are scheduled for organ transplantation, long-term anticoagulation or nonsteroidal anti-inflammatory drug therapy for arthritis and those with cancer of the head and neck.	
G. Familial adenomatous polyposis syndromes.	
H. For confirmation and specific histologic diagnosis of radiologically demonstrated lesions:	
1. Suspected neoplastic lesion.	
2. Gastric or esophageal ulcer.	
3. Upper tract stricture or obstruction	
I. GI bleeding:	
1. In patients with active or recent bleeding.	
2. For presumed chronic blood loss and for iron deficiency anemia when the clinical situation suggests an upper GI source or when colonoscopy does not provide an explanation.	
J. When sampling of tissue or fluid is indicated.	
K. Selected patients with suspected portal hypertension to document or treat esophageal varices.	
L. To assess acute injury after caustic ingestion.	
M. To assess diarrhea in patients suspected of having small-bowel disease (e.g., celiac disease).	
N. Treatment of bleeding lesions, such as ulcers, tumors, vascular abnormalities (e.g., electrocoagulation, heater probe, laser photocoagulation, or injection therapy).	
O. Removal of foreign bodies.	
P. Removal of selected lesions.	
Q. Placement of feeding or drainage tubes (e.g., peroral, percutaneous endoscopic gastrostomy, percutaneous endoscopic jejunostomy).	
R. Dilation and stenting of stenotic lesions (e.g., with transendoscopic balloon dilators or dilation systems using guide wires).	
S. Management of achalasia (e.g., botulinum toxin, balloon dilation).	
T. Palliative treatment of stenosing neoplasms (e.g., laser, multipolar electrocoagulation, stent placement).	
U. Endoscopic therapy of intestinal metaplasia.	
V. Intraoperative evaluation of anatomic reconstructions typical of modern foregut surgery (e.g., evaluation of anastomotic leak and patency, fundoplication formation, pouch configuration during bariatric surgery).	
W. Management of operative complications (e.g., dilation of anastomotic strictures, stenting of anastomotic disruption, fistula, or leak in selected circumstances).	
EGD is generally not indicated for evaluating:	
A. Symptoms that are considered functional in origin (there are exceptions in which an endoscopic examination may be done once to rule out organic disease, especially if symptoms are unresponsive to therapy or symptoms recur that are different in nature from the original symptoms).	
B. Metastatic adenocarcinoma of unknown primary site when the results will not alter management.	
C. Radiographic findings of:	
1. Asymptomatic or uncomplicated sliding hiatal hernia.	
2. Uncomplicated duodenal ulcer that has responded to therapy.	
3. Deformed duodenal bulb when symptoms are absent or respond adequately to ulcer therapy.	
Sequential or periodic EGD may be indicated for:	
A. Surveillance for malignancy in patients with premalignant conditions (e.g., Barrett’s esophagus, polyposis syndromes, gastric adenomas, tylosis, or previous caustic ingestion).	
Sequential or periodic EGD is generally not indicated for:	
A. Surveillance for malignancy in patients with gastric atrophy, pernicious anemia, fundic gland or hyperplastic polyps, gastric intestinal metaplasia, or previous gastric operations for benign disease.	
B. Surveillance of healed benign disease, such as esophagitis and gastric or duodenal ulcer.	

GI: Gastrointestinal; EGD: Esophagogastroduodenoscopy

## MATERIALS AND METHODS

A prospective study was carried out between December 2013 and 2014 in an “open access” endoscopy service in New Plymouth, New Zealand, for general practitioners (GPs) and hospital-based consultants. The service accounts for almost all endoscopies done in this region with very limited private stuff, which is not a part of this study. The OGDs were performed by experienced endoscopists, and the referring physicians were unaware of the study. By agreement among the endoscopists, endoscopic findings were reported according to the internationally accepted terminology and definitions (e.g., Los Angeles classification for esophagitis, etc) in a specially designed software called provation. Data were collected by an assigned surgical registrar on a premade questionnaire, which looked at the following information: (a) Determination of the indication category according to the ASGE guidelines,^9^ based on information provided in the medical documents; (b) urgency and source of referral; (c) findings of the OGD selected from a predefined list in Provation; (d) to determine if the finding is clinically significant or not.

The ASGE guidelines were used to classify the indication into two groups, namely appropriate (generally indicated) and inappropriate (generally not indicated) as shown in Table 1. The endoscopic diagnosis was classified as either clinically significant or insignificant.

If a patient had more than one endoscopic diagnosis, the most severe one was used for the statistical analysis. The OGD was performed irrespective of the indication’s appropriateness. To assess the association between appropriateness and the presence of a clinically significant lesion, patients with ASGE indication for OGD were compared with those in whom an ASGE indication was absent. This association was expressed as odds ratio (OR) of finding a relevant diagnosis in patients with an “appropriate” indication compared with those with a “not appropriate” indication. The association was considered statistically significant if the 99% confidence interval (CI) of the OR did not include the value 1.0. Calculation of the 99% CI was required as multiple tests for statistical analysis were used. The ability of the ASGE indications to predict a relevant endoscopic diagnosis was assessed by calculating the likelihood ratio (positive and negative) both in general and for each indication individually. Differences were only considered significant with p-value less than 0.01 or 1% probability level.

## RESULTS

A cohort of 1,019 consecutive patients referred for OGD were included in this study. Of these, 454 were referred by GPs and 565 by hospital-based consultants; 461 were males and 558 were females. The mean and median ages were 62.6 and 66 respectively. Of the 1,019 procedures performed, 406 (40%) were done acutely and remaining 613 were booked electively. Esophagogastroduodenoscopy was the first examination for 903 (88.6%) patients and was a follow-up exam for 116 (11.4%) patients; 353 (34.6%) referrals were from inpatients, whereas 666 (65.4%) were outpatient referrals both from outpatient clinics and GPs.

Demographic and clinical characteristics of the patients according to the specialty of referring physician is shown in Table 2. The GPs are more likely to schedule examinations for “upper abdominal symptoms that persist despite an appropriate trial of therapy” (8.8% *vs* 15.4%; p < 0.05), “upper abdominal symptoms associated with other symptoms or signs suggesting structural disease or new-onset dyspepsia in patients older than 50 years of age” (5.3% *vs* 11%; p < 0.01), “dysphagia or odynophagia” (11.9% *vs* 3.5%; p < 0.01), “persistent vomiting of unknown cause” (1.8% *vs* 6.6%; p < 0.01), and for “symptoms considered function” (10.8% *vs* 24.7%; p < 0.01). However, examinations for “active or recent GI bleeding” (23.2% *vs* 0%; p < 0.01), “histologic assessment of a neoplasia detected radiologically (1.8% *vs* 0%; p < 0.01), and “surveillance of healed benign lesions (30.6% *vs* 11.2%; p < 0.01) were more frequently requested by the hospital-based consultants.

**Table 2:** **Table 2:** Demographic and clinical characteristics of the study population according to the specialty of the referring physician

		*Hospital-based consultants (n = 565)*		*GPs (n = 454)*	
Sex, M/F		329/236		132/322	
Age, mean ± SD		61.1 ± 17.3		60.3 ± 17.5	
Main referral indication (%)					
• Appropriate indication					
1. Upper abdominal symptoms persistent despite therapy		8.8		15.4*	
2. Upper abdominal symptoms associated with symptoms and signs suggesting serious organic disease or in patients aged >45 years		5.3		11*	
3. Esophageal reflux symptoms persistent or recurrent despite therapy		1.8		4.4	
4. Portal hypertension evaluation		0.9		0	
5. Active or recent GI bleeding		23.2		0*	
6. Suspected chronic bleeding		3.5		6.6	
7. Dysphagia or odynophagia		3.5		11.9*	
8. GI assessment in other medical disorders		1.8		2.2	
9. Persistent vomiting of unknown cause		1.8		6.6*	
10. Sclerotherapy or variceal bleeding		5		0	
11. Histologic assessment of a neoplasia detected radiologically		1.8		0*	
12. Others		1.8		3.5	
• Not appropriate indications					
1. Symptoms considered functional		10.8		24.7*	
2. Surveillance of healed benign lesions		30.6		11.2*	
3. Others		3.5		2.4	

*p < 0.01; GI: Gastrointestinal

The indication for OGD was classified as appropriate, according to the ASGE criteria, in 58% of the cases and not appropriate in 42% of the cases. The frequency of the most common appropriate and inappropriate indications and both positive and negative likelihood ratios for these indications is shown in Table 3. The most frequent appropriate indications in order of frequency were “active or recent GI bleeding” (12.9%); “upper abdominal symptoms persistent, despite an appropriate trial of therapy” (11.8%); and “upper abdominal symptoms associated with other symptoms or signs suggesting structural disease or new-onset dyspepsia in patients older than 50 years of age” (7.9%). On the contrary, the most common inappropriate indications were “surveillance of healed benign lesions like peptic ulcer or esophagitis” (22%) and “symptoms that are considered functional in origin” (17%).

**Table 3:** **Table 3:** Referral indication for OGD and diagnostic yield according to ASGE guideline criteria

*ASGE indication of referral*		*Overall, % (n = 1,019)*		*Relevant lesion, %*		*LR+*		*LR–*	
• Appropriate indication									
1. Upper abdominal symptoms persistent despite therapy		11.8		66.7		1.91		0.92	
2. Upper abdominal symptoms associated with symptoms and signs suggesting serious organic disease or in patients aged >45 years		7.9		91.3		9.96		0.87	
3. Esophageal reflux symptoms persistent or recurrent despite therapy		2.9		76.7		3.14		0.96	
4. Portal hypertension evaluation		0.5		100		10.51		0.99	
5. Active or recent GI bleeding		12.9		53.4		1.09		0.98	
6. Suspected chronic bleeding		4.9		56		1.21		0.99	
7. Dysphagia or odynophagia		7.3		52.7		1.06		0.99	
8. GI assessment in other medical disorders		2.0		55		1.16		0.99	
9. Persistent vomiting of unknown cause		3.9		80		3.82		0.95	
10. Sclerotherapy or variceal bleeding		0.5		100		10.51		0.99	
11. Histologic assessment of a neoplasia detected radiologically		1.0		60		1.43		0.99	
12. Others		2.6		46.2		0.81		1	
13. Total		58		65		1.77		0.45	
• Not appropriate indications									
1. Symptoms considered functional		17		38.7		0.64		1.32	
2. Surveillance of healed benign lesions		22		25.9		0.33		1.33	
3. Others		3.0		38.7		0.64		1.01	
4. Total		42		32		0.45		1.77	

GI: Gastrointestinal

A relevant endoscopic finding was detected in 521 cases (51.4%), whereas not a relevant finding and a normal examination were seen in 15.7 and 32.9% cases respectively. The diagnostic yield was higher for generally indicated OGDs compared with those described as not indicated (65% *vs* 32%; OR 3.94, 99% CI 2.785, 5.575; p < 0.01). Overall, likelihood ratio (LR)^+ve^ and LR^–ve^ of appropriate indications for detecting a relevant lesion were 1.77 and 0.45 respectively.

The most frequent relevant and not relevant endoscopic findings are listed in Table 4. The most frequent relevant findings were peptic ulcer disease [118 patients (11.5%)], erosive gastritis [113 patients (11%)], and erosive esophagitis [108 patients (10.5%)]. Malignancies were detected in 1.6% of the cases. None of the cancers were diagnosed in patients judged to have not appropriate indication for OGD. Barrett’s esophagus was found more frequently in patients with no appropriate indications for OGD [14 *vs* 3, OR 0.15; 99% CI (0.029–0.78), p < 0.01].

**Table 4:** **Table 4:** Main endoscopic findings according to appropriateness of the indication

*Endoscopic finding*		*Not appropriate indications, n (%)*		*Appropriate indications, n (%)*		*OR (99% CI)*	
Clinically relevant							
1. Erosive esophagitis		17 (4)		91 (15.4)		4.40 (2.18–8.87)*	
2. Erosive gastritis		46 (10.7)		67 (11.3)		1.06 (0.63–1.79)	
3. Esophageal varices		0 (0)		10 (1.7)		15.47 (0.37–645.5)	
4. Duodenal ulcer		9 (2.1)		91 (15.4)		8.47 (3.39–21.17)*	
5. Barrett’s esophagus		14 (3.3)		3 (0.5)		0.15 (0.029–0.78)*	
6. Gastric ulcer		0 (0)		18 (3)		27.64 (0.68–1111.64)	
7. Erosive duodenitis		37 (8.6)		66 (11.2)		1.32 (0.76–2.31)	
8. Gastric polyps		16 (3.7)		13 (2.2)		0.57 (0.21–1.53)	
9. Gastric cancer		0 (0)		7 (1.2)		10.99 (0.25–474.45)	
10. Esophageal cancer		0 (0)		10 (1.7)		15.47 (0.37–645.55)	
11. Esophageal stenosis		6 (1.4)		3 (0.5)		0.35 (0.05–2.23)	
Not clinically relevant							
1. Normal		246 (57.4)		89 (15)		0.13 (0.08–0.19)*	
2. Nonerosive gastritis		12 (2.8)		65 (11)		4.28 (1.87–9.79)*	
3. Non erosive duodenitis		0 (0)		6 (1)		9.51 (0.21–417.96)	
4. Hiatus hernia		25 (5.8)		52 (8.8)		1.55 (0.81–2.97)	
Total		428		591			

*p < 0.01

## DISCUSSION

Upper GI endoscopy is a safe and accurate procedure for diagnosing conditions like peptic ulcer disease, upper GI malignancies, celiac disease, etc.^10^ Hence, an open access system was adopted. Since its application, there has been a steady increase in the number of OGDs,^1,11^ resulting in an increase in the waiting times and overloaded waiting lists, with risk of delaying the exam for patients with potentially severe disease.^12^ Therefore, evaluation of both the appropriateness and the diagnostic yield in relation to each clinical indication is critical to the assessment of the costs and benefits of procedures performed in an open access setting.^13^ Rate of inappropriate indications for OGD has been found to be ranging from 5 to 49% in different studies.^2,14^

There was not much difference in the rate of inappropriate referrals between the GPs and the hospital-based consultants. The GPs sent more referrals with persistent abdominal pain despite medical treatment, dysphagia, and persistent vomiting. On the contrary, hospital-based consultants referred patients mostly for GI bleeding and histologic assessment of a lesion detected radiologically.

The effectiveness of an open access system is judged by the diagnostic yield of the OGDs, which is the ability to detect a clinically significant lesion important for patient care. However, a normal examination though regarded as not significant, in some cases, is helpful to the patient’s care by ruling out a serious condition. In the present study, clinically relevant lesions were found in just over half of the patients. We did not include in our study the histologic assessment of apparently normal mucosa which in some cases can diagnose conditions like celiac disease and increase the yield of clinically significant lesions. Furthermore, the inclusion of gastric polyps in clinically significant lesion list can be questioned. The probability of finding a clinically relevant lesion was higher when OGD was performed for an appropriate compared with an inappropriate indication (indeed twofold higher). Such a difference appeared to be mainly referred to the low LR (LR^–^, 0.45) of a relevant finding when an OGD is judged inappropriate, being only slightly raised when an appropriate indication was present (LR^+^, 1.77).

Importantly, the OR for neoplastic lesions was very high; none of them were diagnosed in indications judged to be not appropriate. However, to our surprise, Barrett’s disease was diagnosed more frequently among patients with not appropriate indications, which are of concern as it is a premalignant condition and different treatment options are available if diagnosed early. There is controversy in the literature about this as some studies suggest that most of the cancers are diagnosed in patients having appropriate indications,^8^ whereas others found a substantial proportion of cancers among not appropriate examinations.^5,9^

These results suggest that ASGE guidelines can be used for monitoring the activity of an open access system. In fact, these observations suggest that at least in our setting if open access system is used appropriately, it is less expensive than the OGD with prior clinical consultation. On the other hand, relevant findings although benign were seen in patients with not appropriate indications. Indeed, a third of the patients with an inappropriate indication had a significant finding and that Barrett’s esophagus was found more frequently in indications judged to be not appropriate. Such negative predictive values, not higher than 70%, make the use of these guidelines in the care of individual patients more uncertain. Hence, under no circumstance should the care of individual patients be guided solely by the guidelines without further clinical information.

## CONCLUSION

In summary, this study showed that upper GI endoscopy in the setting of an open access system is a useful procedure. As most of the relevant findings especially neoplastic lesions were found when OGD was performed for appropriate indication, the appropriateness of indication as decided by the ASGE criteria is crucial to the cost-effectiveness of an open access system.

## CLINICAL SIGNIFICANCE

The ASGE guidelines are an effective tool in selecting patients likely to have pathology on OGD, and hence, patients who are in need of OGD get the examination without being delayed by patients who are likely not to have a serious pathology. However, these guidelines should not be solely relied on in selecting patients, and selection should be a combination of these guidelines and the clinical context.
